# Conservation Implications of Shifting Gut Microbiomes in Captive-Reared Endangered Voles Intended for Reintroduction into the Wild

**DOI:** 10.3390/microorganisms6030094

**Published:** 2018-09-12

**Authors:** Nora Allan, Trina A. Knotts, Risa Pesapane, Jon J. Ramsey, Stephanie Castle, Deana Clifford, Janet Foley

**Affiliations:** 1 Department of Medicine and Epidemiology, School of Veterinary Medicine, University of California, Davis, CA 95616, USA; neallan@ucdavis.edu (N.A.); rrpesapane@ucdavis.edu (R.P.); stcastle@ucdavis.edu (S.C.); Deana.Clifford@wildlife.ca.gov (D.C.); 2 Wildlife Investigations Lab, California Department of Fish and Wildlife, 1701 Nimbus Road, Rancho Cordova, CA 95670, USA; 3 Department of Molecular Biosciences, University of California, Davis, CA 95616, USA; taknotts@ucdavis.edu (T.A.K.); jjramsey@ucdavis.edu (J.J.R.)

**Keywords:** Amargosa vole, captive breeding, *Microtus californicus scirpensis*, diet, foregut, feces, microbiota

## Abstract

The Amargosa vole is a highly endangered rodent endemic to a small stretch of the Amargosa River basin in Inyo County, California. It specializes on a single, nutritionally marginal food source in nature. As part of a conservation effort to preserve the species, a captive breeding population was established to serve as an insurance colony and a source of individuals to release into the wild as restored habitat becomes available. The colony has successfully been maintained on commercial diets for multiple generations, but there are concerns that colony animals could lose gut microbes necessary to digest a wild diet. We analyzed feces from colony-reared and recently captured wild-born voles on various diets, and foregut contents from colony and wild voles. Unexpectedly, fecal microbial composition did not greatly differ despite drastically different diets and differences observed were mostly in low-abundance microbes. In contrast, colony vole foregut microbiomes were dominated by *Allobaculum* sp. while wild foreguts were dominated by *Lactobacillus* sp. If these bacterial community differences result in beneficial functional differences in digestion, then captive-reared Amargosa voles should be prepared prior to release into the wild to minimize or eliminate those differences to maximize their chance of success.

## 1. Introduction

The Amargosa vole (*Microtus californicus scirpensis*) is a highly endangered rodent endemic to a small stretch of the California portion of the Amargosa River basin in the Mojave Desert near Tecopa in Inyo County [1,2]. The patchily distributed habitat for Amargosa voles features permanent water and vegetation dominated by Olney’s three-square bulrush (*Schoenoplectus americanus*), which is the primary food source for this species [3]. The marshes used by voles are surrounded by harsh desert, creating a structured metapopulation of small subpopulations [4]. Land development and decreased availability of water further reduced existing habitat [2,4] and the Amargosa vole was listed as endangered by the state of California [5] and by the US Fish and Wildlife Service [6]. In response to severe habitat degradation available to one of the previously most sustainable subpopulations [7], a captive breeding population was established at the University of California, Davis in July 2014. The colony originated from 20 wild-caught juvenile Amargosa voles removed from the Amargosa River basin and serves as an insurance colony and as a source of individuals to release into the wild as restored habitat becomes available.

While the colony is highly successful with vole breeding and husbandry, releases of captive-reared animals back into the wild have not been successful to date [8]. Releases of captive-reared animals are notoriously challenging for a variety of reasons, including stress of release [9], lack of wild-type behaviors in captive-reared stock [9,10], competition with wild conspecifics [11,12,13], adaptations suited to captivity [14,15], disease [16,17], and difficulty identifying and transitioning to a native diet [9,13,18]. Indeed for the Amargosa vole, transitioning to a diet of native foods is a major concern. To ensure the survival and balanced nutrition of colony voles, they were successfully transitioned to a diet of Teklad 2018 (Envigo, Hayward, CA, USA) commercial rodent chow [19] and later higher-fiber rabbit chow (LabDiet 5326–3, LabDiet, St. Louis, MO, USA). The colony has been maintained on these commercial diets for multiple generations. However challenges during captive-release attempts revealed potential pitfalls of their diet in captivity. Released voles failed to eat and some subsequently suffered from hepatic lipidosis. When efforts were made to transition captive-reared animals to a wild diet in captivity, they were unable to maintain a healthy weight and body condition. These findings prompted investigation into factors affecting the ability of captive-reared voles to extract sufficient nutrition from their native diet.

Bulrush is extremely low in nutrients and high in fiber, making it likely that wild Amargosa voles need specialized digestive systems to sustain themselves on an all-bulrush diet [20,21]. Like other species with low quality diets, the Amargosa vole likely depends on gut microbes to ferment dietary components and maximize the nutrition they receive from otherwise unavailable energy sources in bulrush [22,23,24,25,26,27]. However life in captivity can result in gut microbiome changes [28,29,30,31]. Wild individuals brought into captivity may retain most of their initial gut microbial composition [32], but animals reared in captivity tend to possess less diverse gut microbiomes than their wild counterparts [28] and dietary specialist species may be even more susceptible to diversity loss [33]. This has led to concern that captive-reared Amargosa voles raised on a captive diet may no longer harbor essential plant-fermenting bacteria necessary to digest bulrush, potentially affecting the success of future release attempts.

To explore the relationship between host diet and gut microbial composition, we analyzed feces from voles on three different diets (colony, colony-transitional, and wild-captured) as well as foregut contents from voles on two different diets (colony and wild). We aimed to characterize the fecal and foregut microbiota of the endangered Amargosa vole to determine how diet in captivity may influence microbial composition and inform captive animal management for effective conservation.

## 2. Materials and Methods

### 2.1. Animals

All work involving live Amargosa voles conformed to guidelines for humane use of animals in research and was approved by the UC Davis Institutional Animal Care and Use Committee (protocol 19741) and was authorized by U.S. Fish and Wildlife Service endangered species recovery permit TE54614A-1.

Seven captive-reared adult male Amargosa voles were included in the study, comprising four sampled for feces and three for foregut contents. No animals were siblings, nor had received prior medications [34,35]. Voles were individually-housed in an indoor animal facility as described by Allan et al. [8]. Voles sampled for feces were from the second colony generation and were fed Teklad 2018 commercial rodent chow (Envigo, Hayward, CA, USA) ad libitum from weaning, comprising the ‘colony’ diet. Voles were noted to consume approximately four pieces of chow per day (~18 g). After baseline colony diet fecal sampling, the four voles were switched to the ‘colony-transitional diet’. Their diet was altered over two weeks by gradually reducing rodent chow to two pieces per day (~9 g) and providing greenhouse-grown bulrush in a cup of potting soil every 5 days. Nutrient comparisons between greenhouse-grown and wild bulrush plants can be found in Supplementary Table S1. After the two-week adjustment period, voles were maintained on the colony-transitional diet for 24 days and their feces sampled again. Voles sampled for foregut contents were from the third colony generation and were fed LabDiet 5326 commercial rabbit chow (manufactured by LabDiet, St. Louis, MO and milled into larger pieces by Stewart’s Feed Service INC, Lawrenceville, GA) ad libitum from weaning, comprising their ‘colony diet’. By the third colony generation, all animals had been transitioned to this rabbit chow to better mimic a natural high-fiber vegetation diet. A comparison of nutrient content in Teklad 2018 rodent chow and LabDiet 5326 rabbit chow can be found in Supplementary Table S2. All three voles sampled had developed early stages of molar apical elongation [36] and required euthanasia, which allowed for opportunistic sampling of foregut contents. Although severe molar apical elongation can inhibit food intake when incisor malocclusion occurs, no malocclusion or weight loss was observed in the individuals sampled.

Seven wild-born Amargosa voles were included in the study, comprising four sampled for feces (all male) and three sampled for foregut contents (one male and two females). All were captured in Tecopa Hot Springs, Inyo Country, California from the same site that sourced founders for the captive colony. Trapping was conducted using Sherman traps baited with a mixture of peanut butter and oats; traps were opened at night and animals collected at first daylight. Animals were considered adult based on body mass, coat color, and testicular status. Wild-born voles used for fecal sampling were transferred to a nearby mobile laboratory with light cycles controlled to mimic natural conditions. They were individually housed in polycarbonate cages with wire lids and furnished with water and bulrush litter at least 10 cm deep. Native bulrush, slices of carrot, sweet potato, apple, jicama, and Teklad 2018 chow were provided. The variety of foods provided was to encourage feeding despite the stress of capture. Animals were noted to have consumed most or all of the native bulrush provided and nibbled on the other food types present. Fecal samples were collected between 24–48 h after transfer to the mobile laboratory. Individuals sampled for the foregut microbiome were collected either after inadvertent trap mortality or euthanasia for illness (such as poor body condition, injury, or heavy ectoparasite load) allowing opportunistic collection of foregut samples. A summary of these diet groups is provided in Table 1.

### 2.2. Sample Collection

For fecal sample collection, each vole was moved from its home cage into a clean cage without bedding. After 15 min, the vole was removed and fecal pellets were collected using a fresh glove, excluding any feces that appeared to have been stepped on by the vole or come into contact with urine. Samples were placed into dry, sterile screw-top tubes and immediately buried in wet ice for transfer to a −80 °C freezer within 30 min (colony samples) or in dry ice (field samples).

For foregut sample collection, animals were euthanized with ketamine and xylazine followed by barbiturate euthanasia solution and cervical dislocation. Carcasses were dissected immediately after euthanasia or within 1 h of discovered trap mortality. Contents were collected from the foregut, which we defined as the proximal segment of the stomach. Samples were placed into screw-top tubes and immediately frozen in a −80 °C freezer (colony samples) or liquid nitrogen (field samples). The only exception was one wild sample, in which the entire digestive system was frozen in liquid nitrogen and briefly thawed to collect foregut contents at a later time.

### 2.3. Sample Analysis

Fecal and foregut samples were submitted through the University of California, Davis Mouse Biology Program for analysis using the Illumina sequencing platform. All fecal samples were submitted at the same time and all foregut samples were submitted at the same time to avoid potential run-to-run variation. Samples were shipped on dry ice to Molecular Research (Lubbock, TX, USA) for total DNA extraction using a PowerSoil kit (Qiagen, Valencia, CA, USA). Foregut samples thawed in transit but a large number of high quality reads were still received. 16S rRNA gene V3-V4 variable region PCR primers 515/806 with barcode on the forward primer were used in a 30 cycle PCR using the HotStarTaq Plus Master Mix Kit (Qiagen, Hilden, Germany) under the following conditions: 94 °C for 3 min, followed by 28 cycles of 94 °C for 30 s, 53 °C for 40 s and 72 °C for 1 min, after which a final elongation step at 72 °C for 5 min was performed. After amplification, PCR products were examined by agarose gel electrophoresis for success of amplification and relative band intensity. Multiple samples were pooled together in equal proportions (based on their molecular weight and DNA concentrations), purified using calibrated Ampure XP beads, and then used to prepare a DNA library using the Illumina TruSeq DNA library preparation protocol. Sequencing was performed at Molecular Research (www.mrdnalab.com, Shallowater, TX, USA) on a MiSeq following the manufacturer’s guidelines. The raw sequence reads from this study were uploaded to NCBI’s Sequence Read Archive under SRP151365. To assess contamination from kit reagents, we ran 2 blank extractions (DNA free water extracted) with each of the kits used in this study. Library PCR did not generate appreciable signal, and no sequence was generated.

The Q25 sequence data derived from sequencing was processed using a proprietary analysis pipeline of MR DNA. In summary, sequences were joined, depleted of barcodes, and then sequences <150 bp or with ambiguous base calls removed. Sequences were denoised, operational taxonomic units (OTUs) generated, and chimeras removed. OTUs were defined by clustering at 3% divergence (97% similarity). Final OTUs were taxonomically classified using BLASTn against a curated database derived from GreenGenes, RDPII, and NCBI [37]. The readmap.uc file was converted to a QIIME-compatible OTU table using usearch [38] and a phylogenetic tree was created. The taxonomy was then added to the biom formatted OTU table. This file was used for analysis of alpha and beta diversity analysis in QIIME 1.9 [39].

Alpha and beta diversity measures were calculated using QIIME 1.9.1. To account for differences in the number of reads in a sample, we performed rarefaction to collect a random set of sequences on a set number of sequences to rule out sample depth bias. The sampling depth was set at 46,000 sequences per sample. Six different measures of alpha diversity were analyzed (Chao1, Good’s coverage, observed species, Faith’s phylogenetic diversity (PD), Shannon index, and Simpson’s index). Beta diversity was evaluated using principal coordinates analyses based on Bray-Curtis distances, which provides a non-phylogenetic measure of community composition differences. Low abundance taxa (less than 0.05%) were filtered from the data set before subsequent statistical analyses.

### 2.4. Statistical Analysis

Differences in abundances between groups were analyzed using one-way ANOVA and the TUKEY test. Differences in fecal microbial abundances before and after captive-reared voles were switched to the captive-transitional diet were analyzed using a paired *t*-test. A two-sided *p* value of 0.05 was considered significant. Community profiles were visualized with Krona charts [40]. Core microbiota for each experimental group was considered to be the set of all OTUs that were present in all samples, and were compared among groups using Venn diagram analyses [41] to visualize overlapping and group specific taxa. A PERMANOVA was used to determine statistical significance of beta diversity.

Partial least squares-discriminant analyses (PLS-DA) were performed on genus-level microbial abundance data using the package mdattools [42] in R [43].

## 3. Results

### 3.1. Fecal Microbiome Analysis

**Figure 1:**
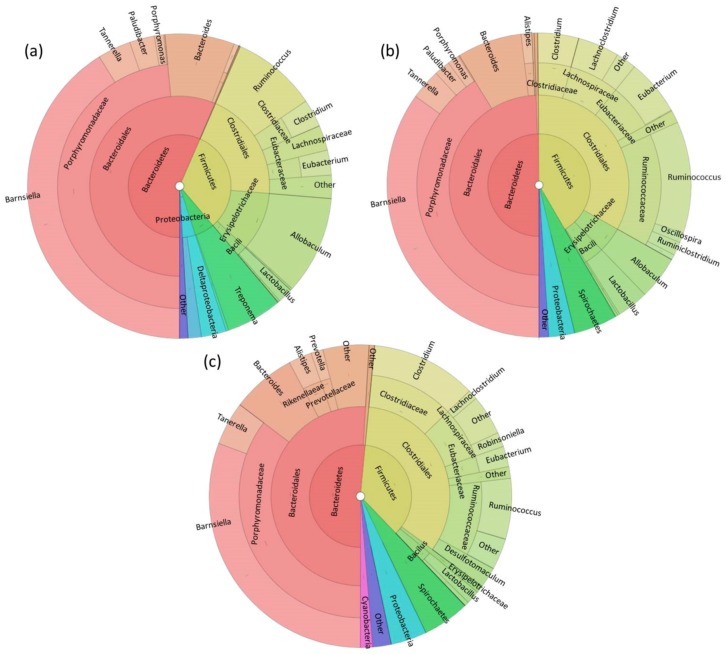
Cladogram-style pie charts (Krona charts) showing the average relative abundance of taxa in (**a**) colony diet, (**b**) colony-transitional diet, and (**c**) wild-captured diet fecal samples. All ‘other’ categories in each chart encompass taxa present at <1% abundance.

**Figure 2:**
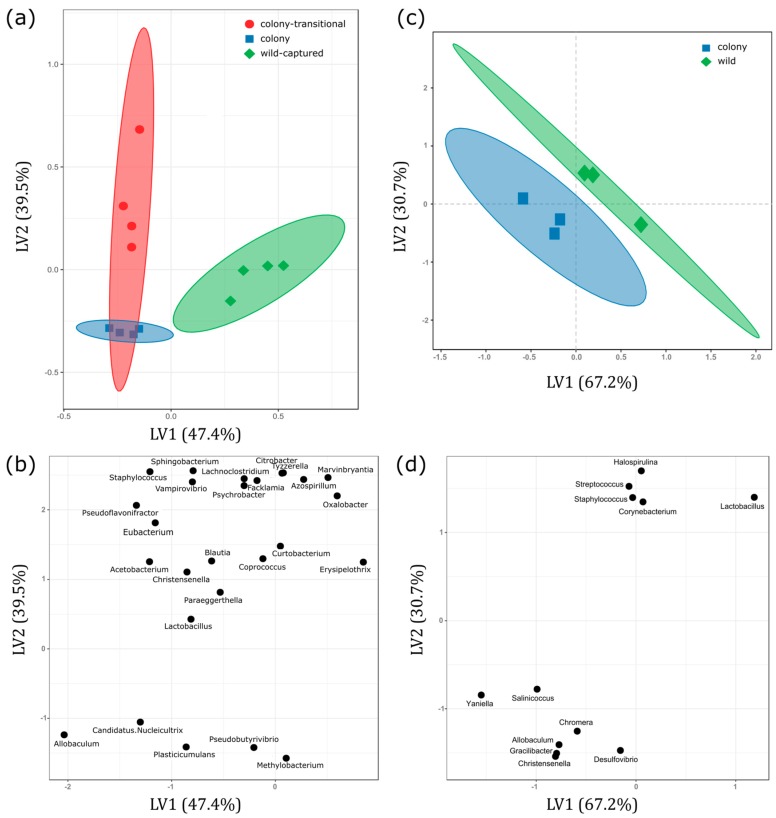
Partial least squares-discriminant analysis (PLS-DA) reveals discriminating characteristics of genus-level microbiota depending on diet group. Figures display (**a**) scores plot of fecal samples (**b**) PLS-DA loadings scatterplot of fecal samples (**c**) scores plot of foregut samples and (**d**) PLS-DA loadings scatterplot of foregut samples. Scores plots cluster based on group assignment. Each symbol represents an individual vole. Shaded ellipses indicate 95% confidence intervals. PLS-DA loadings scatterplots show the genera that influence how groups are being pulled apart. PLS-DA loadings are limited to VIP scores >2.

Eleven phyla were identified in fecal samples with no significant differences in relative abundance between colony, colony-transitional, and wild-captured diet groups. All samples were dominated by Bacteroidetes and Firmicutes (Figure 1, Supplementary Table S3). The most dominant taxon at each taxonomic level was the same for all diet groups, although there were significant differences observed in lower taxonomic levels and low-abundance taxa (<1%) (Supplementary Tables S3–S7). When captive-reared voles were switched from the colony diet to the colony-transitional diet, unexpectedly there were few shifts in relative abundance and any that were significantly different were in low abundance taxa (<5%). Most significant differences were noted when comparing wild-captured and colony samples. We performed PLS-DA using genus-level abundance data to investigate signatures related to each diet. PLS-DA discriminated genera based on VIP (variable importance in projection) scores showing which taxa contributed most to separation of the diet groups as shown by score and loadings scatterplots (Figure 2a,b; Supplementary Table S8). Colony and colony-transitional samples did slightly cluster away from wild-captured samples. Based on VIP scores and the five most important genera for discriminating the diet groups were *Erysipelothrix*, *Sphingobacterium*, *Acetobacterium*, *Azospirillum,* and *Vampirovibrio*, all of which were present in low abundances (Supplementary Table S8).

**Figure 3:**
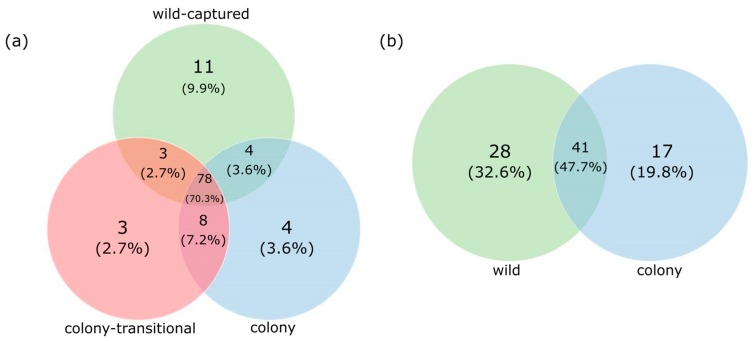
Venn diagram of the core bacterial communities showing the number of shared and unique bacterial taxa in (**a**) fecal samples and (**b**) foregut samples. OTU presence was required in all samples within a diet group in order to be classified as core. OTUs include taxa present at very low abundances (<0.5%).

Based on a Venn diagram comparing core gut microbiota in colony, colony-transitional, and wild-captured fecal samples, including very low-abundance taxa, 70.3% of OTUs were shared by all diet groups (Figure 3a). When filtered to exclude taxa present at less than 0.05% abundance, 96.2% of OTUs were shared by all diet groups in the feces (Supplementary Figure S1a).

**Figure 4:**
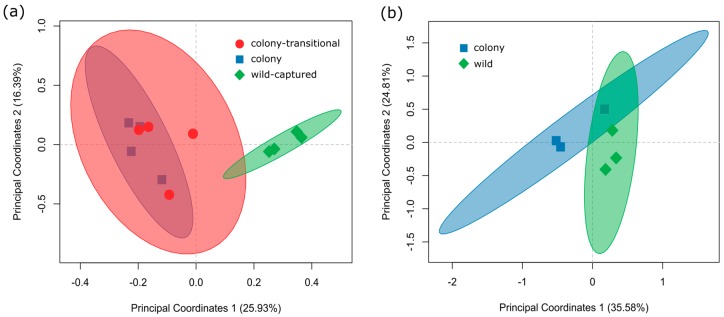
PCoA based on Bray-Curtis distances was used to assess microbial beta diversity of (**a**) colony, colony-transitional, and wild-captured fecal samples and (**b**) colony and wild foregut samples. Each symbol represents an individual vole. The distances between sample points represent the similarity of microbiota in the samples. Closer distances represent higher similarity of the samples. The shaded ellipse depicts the 95% confidence interval.

Alpha diversity metrics (Chao1, Faith’s whole tree phylogenetic diversity, Good’s coverage, observed species, Shannon, Simpson’s) did not significantly differ between the colony, colony-transitional, and wild-captured diet groups (Supplementary Table S9). A PERMANOVA based on Bray-Curtis distances confirmed that wild-captured samples significantly (*p* = 0.003) clustered away from colony and colony-transitional samples (Figure 4a). A PERMANOVA based on unweighted UniFrac distances to account for phylogenetic relatedness showed similar significant results (*p* = 0.006).

### 3.2. Foregut Microbiome Analysis

**Figure 5:**
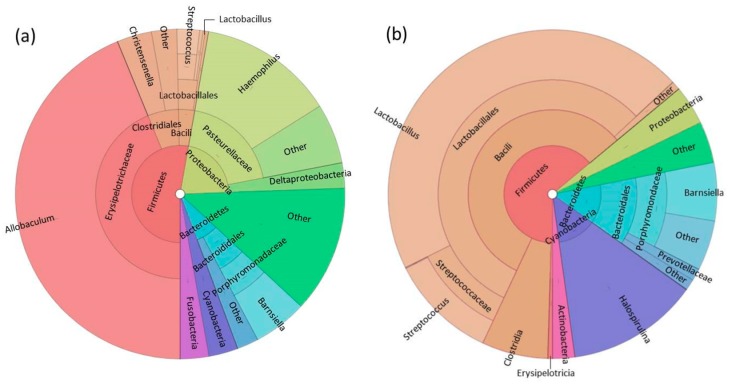
Cladogram-style pie charts (Krona charts) showing the average relative abundance of taxa in (**a**) colony and (**b**) wild foregut samples. All ‘other’ categories in each chart encompass taxa present at <1% abundance.

Fourteen phyla were identified in foregut samples with no significant differences in relative abundance between colony and wild diet groups. Firmicutes was the dominant phylum in both groups (Figure 5, Supplementary Table S10). Only two significant differences in relative abundance were identified by ANOVA and Tukey post-tests at any taxonomic level: One in the class Bacilli and one in the order Lactobacillales, which is within the Bacilli class. Both were significantly more abundant in wild samples. Lactobacillales made up 56.2 ± 16.43% of microbial abundance in wild individuals compared to only 3.09 ± 2.53% in colony individuals (*p* = 0.033). Though no other differences in relative abundance reached statistical significance, wild and colony samples were consistently dominated by different taxa at every taxonomic level below phylum (Supplementary Tables S10–S14). Class Erysipelotrichia and its lower-lower level taxa (order: Erysipelotrichales, family: Erysipelotrichaceae, genus: *Allobaculum*) dominated colony samples while class Bacilli and its lower-level taxa (order: Lactobacillales, family: Lactobacillaceas, genus: *Lactobacillus*) dominated wild samples (Figure 5, Supplementary Table S14). We performed PLS-DA using genus-level abundance data to investigate signatures related to each group as shown by score and loadings plots (Figure 2c,d). Wild and colony samples did segregate from each other. Based on VIP scores, the five most important genera for discriminating the groups were *Desulfovibrio*, *Allobaculum*, *Gracilibacter*, *Christensenella*, and *Lactobacillus* (Supplementary Table S15).

A Venn diagram comparing core gut microbiota present in colony and wild fecal samples. When data were unfiltered to include all taxa, including extremely low-abundance taxa, 47.7% of OTUs were shared by both diet groups (Figure 3b). When filtered to exclude taxa present at less than 0.05% abundance, 65.2% OTUs were shared by all diet groups in the foregut (Supplementary Figure S1b).

Alpha diversity metrics (Chao1, Faith’s whole tree phylogenetic diversity, Good’s coverage, Observed Species, Shannon, Simpson’s) were not significantly different between wild and colony foregut samples (Supplementary Table S16). Beta diversity did not significantly differ between wild and colony samples (Figure 4b).

## 4. Discussion

This study presents a rare comparison of the gut microbiomes of captive-reared endangered animals to wild and recently captured conspecifics from the founder population. Fecal microbiome analysis revealed differences in composition between diet groups and slight enrichment in fiber-fermenting taxa in wild-captured samples, but otherwise showed unexpectedly similar microbial composition and core microbiota regardless of diet. Foregut microbiome analysis showed fewer statistically significant differences than the feces, however differences in dominant taxa of foregut samples could indicate important functional differences between wild and captive individuals’ ability to maintain weight on a nutrient-poor diet.

Dramatic shifts in fecal microbial abundance due to diet have been well-documented in a variety of species [33,44,45,46,47], particularly when diets differ in fiber content [48,49,50,51,52,53,54]. While we did observe differences between the fecal microbiomes of each diet group, they were less than expected considering the different diets and diet histories of our study animals. All diet groups were dominated by the same taxa at all levels (Figure 1, Supplementary Tables S3–S7). Nearly all significant differences were identified in very low-abundance taxa: It is not known whether some of these may have been transient environmental microbes or contaminants rather than resident bacteria as we did not test microbes in water, soil, or food samples. Half of the eight significantly different genera (*Dehalobacterium*, *Hallela*, *Sphingobacterium*, *Natranaerovirga*) identified were likely from exposure of captive-transitional and wild-captured animals to bacteria from environmental sources such as soil, algae, and wetlands [55,56,57,58,59,60]. Lack of more numerous significant differences could be due to small sample sizes, although this is not likely as there was low variance between fecal samples.

Given the high plant-matter intake of wild-captured voles, both before capture and in captivity, we expected to detect significantly higher abundances of fiber-fermenting bacteria from those individuals. Genera *Clostridium* and *Prevotella,* both of which are associated with high-fiber diets and fermentation [54,61,62,63], were significantly enriched in wild-captured fecal samples and represented 11.6 ± 2.96% and 1.34 ± 0.61% of microbial abundance in wild-captured samples, respectively. No other fiber-fermenting taxa, such as within families Ruminococcaceae and Lachnospiraceae [64,65,66,67], were significantly different between diet groups, possibly indicating that enriched *Clostridium* and *Prevotella* may be important for fiber fermentation in the hindgut of this species while eating mostly bulrush.

Bray-Curtis distances showed that wild-captured samples clustered on their own while colony and colony-transitional samples clustered together (Figure 4a). This is in line with previous studies that show different microbial diversity and community structure in captive vs wild conspecifics [28,48,68,69]. PLS-DA analysis also indicated separation of wild-captured samples (Figure 2a). The five genera that contributed most to PLS-DA separation according to VIP scores were present at very low-abundance in all diets (<0.5%) and are typically found in association with soil, water, plant roots, or algae [70,71,72,73,74] (Supplementary Table S8). This suggests that exposure to environmental wetland features prior to capture is likely an important source of commensal bacteria or host-associated microbiota, thus driving the separation of wild-captured samples from colony and colony-transitional samples in our analyses.

Core fecal microbiome analysis revealed high overlap between all three diet groups. All groups shared 70.3% OTUs when all taxa were accounted for (Figure 3a) and all taxa unique to each diet group were present at very low abundances (>0.05%) (Supplementary Figure S1a). This suggests that while colony and wild-captured voles may show different community structures with shifts in relative abundance, they largely harbor the same microbial taxa. A similar degree of overlap was observed by Kohl et al. [32] who found that wild woodrats shared 68% of their original OTUs with samples taken after being fed commercial chow in captivity for two weeks, and 64% after six months. Though abundances shifted over time, as we also observed in our study, they argue that overall their rodents retained similar composition upon entrance to captivity. While our study did not compare wild-born animals before and after entrance to captivity, the overall sentiment may apply to Amargosa voles as well.

Core taxa shared only by wild-captured and colony-transitional samples were of particular interest to determine if the colony-transitional diet reintroduced any important wild microbes to colony-born individuals. The three core OTUs shared by only those diet groups included *Methanobrevibacter*, *Cytophaga*, and *Ethanoligenens*. *Cytophaga* and *Ethanoligenens* are likely to be transient microbes associated with soil [75,76,77], which both wild-captured and colony-transitional animals were exposed to through their diets. *Methanobrevibacter* has been associated with methane production in termite and bovine hindguts [78,79] and could indicate increased fiber fermentation during vegetation consumption, despite its very low abundance.

Though we cannot consider wild-captured voles a true representation of wild voles due to their brief time in captivity, the similarities between their fecal microbiomes to those of colony and colony-transitional voles are striking. Significant changes in fecal microbiota have been observed within 24 hours of a diet change [52,80] and given that other vole species have mean gut retention times between 6–13.1 h [23,81,82], 24–48 h in captivity likely allowed for some shifts to occur in the wild-captured voles we sampled. However considering that wild-captured voles were noted to consume almost entirely native bulrush while in captivity, it is doubtful that exposure to such small amounts of non-native foods and commercial chow would cause their microbiomes to so closely resemble those of second generation colony-born individuals [32,54]. Either Amargosa voles retain largely resilient hindgut microbiomes that resemble wild founders even after generations in the colony, or wild voles lose much of their unique fecal microbiota almost immediately after being brought into captivity, quickly adapting to a captive diet. The latter possibility would be problematic for conservation purposes, particularly considering that shifts towards natural foods in the form of the colony-transitional diet did not shift colony microbiomes closer to those of recently wild voles.

In contrast to fecal samples, foregut samples showed fewer significant differences between diet groups but hinted at more prominent functional differences. Colony foregut samples were dominated by lower-level taxa of class Erysipelotrichia, specifically genus *Allobaculum*, while wild foregut samples were dominated by lower-level taxa of class Bacilli, specifically genus *Lactobacillus*. Though differences between abundances of genera did not reach statistical significance, PLS-DA analysis and VIP scores highlighted *Allobaculum* and *Lactobacillus* as two of the five most important genera in discriminating captive and wild samples (Figure 2d, Supplementary Table S15). *Allobaculum* species reportedly break down cellulose into lactate and butyrate [83]. *Allobaculum* is not highly abundant in the foregut chambers of other herbivorous rodents, which are instead dominated by *Lactobacillus* [84,85,86]. Dominance of *Lactobacillus* in wild foregut samples was therefore not unexpected. *Lactobacillus* species do not degrade complex polysaccharides and are unlikely to be conducting plant fiber fermentation in the foregut, but are associated with lactic-acid fermentation, increased bile salt hydrolase activity, and fermentation of simple sugars [86,87,88]. There is also evidence that *Lactobacillus* may be associated with efficient nutrient extraction. Kohl et al. [65] observed higher abundances of Lactobacillus in bank voles selected for their ability to maintain weight on a high-fiber herbivorous diet compared to control animals. They suggested that *Lactobacillus* could be important for recycling urea nitrogen in the foregut, thereby improving an animal’s nitrogen economy and allowing it to maintain body mass on a low-protein diet [65,85,89]. If this is the case, high levels of *Lactobacillus* in the foregut of wild Amargosa voles could provide them with an important advantage over colony-reared individuals when attempting to survive on a low-nutrient wild diet. Genera *Gracilibacter*, *Desulfovibrio*, and *Christensenella* were also included in the top five highest VIP scores important for discriminating samples and all were more abundant in colony foreguts. *Gracilibacter* is likely transient environmental bacteria based on its association with wetlands [90]. *Desulfovibrio* and *Christensenella* are common residents of the mammal gut [91,92,93], though *Desulfovibrio* is also associated with mud and water sediments, making it a possible environmental contaminant [94]. High abundances of *Christensenella* in feces is often associated with leanness in humans and rodents [91,95], though its associations in the foregut are not well-studied.

Microbial diversity in the foregut did not significantly differ between the two diets (Figure 4b); however wild and colony samples had far less overlap of core microbiota than fecal samples did, indicating a higher number of taxa unique to each diet group (Figure 3b). The majority of core taxa unique to wild foregut samples were present at very low abundances (<0.05%); however the presence of genera *Lactococcus* and *Methanobrevibacter* in all wild samples could potentially indicate increased digestion of simple sugars and fiber [78,79,96].

With larger sample sizes we would likely have identified more statistically significant differences in abundance and diversity between samples and groups, however small sample size was necessary due to the Amargosa vole’s endangered status and limitations of collection. Fecal sample collection was limited by trapping procedures that would have contaminated feces collected directly from traps and by the number of wild individuals authorized to be held temporarily in captivity before re-release. Foregut samples were further limited and could only be collected from individuals euthanized due to illness or found deceased. The small number of wild animals that met those criteria each year meant we were unable to select for sex of animals sampled for foregut contents. Subtle but significant differences have been identified in the fecal microbiota of female vs male wild mice [97], but the effects of sex on the foregut microbiome are not known. Illness and death of individuals sampled could have affected foregut microbial composition [98,99,100] as after death of the host, gut microbial diversity decreases and richness of resident anaerobic bacteria increases [101,102]. Though this could have contributed to the high abundance of the anaerobe *Lactobacillus* in wild foregut samples, it does not account for the high abundance of *Allobaculum,* which is also an anaerobe, in colony samples that were collected immediately following euthanasia. The remaining anaerobic genera detected were not present at notably different abundances in wild and colony samples, suggesting that the microbiomes of deceased animals included in the study did not undergo immense changes before sample collection.

## 5. Conclusions

Though researching endangered species presents unique challenges and limitations, evaluating factors that contribute to the failure or success of captive releases is imperative for informing appropriate management, release planning, and successful conservation work for captive propagation programs. Despite small sample sizes, our study provides evidence that investigating the gut microbiome is a vital consideration for captive-release preparation for the Amargosa vole. While fecal sampling is a widely-used and non-invasive method of gut microbial analysis, it may not fully capture the complexities and differences in other sections of the gut [85,103]. Our fecal analysis suggests a small but significant increase in two fiber fermenting bacteria as well as differences in beta diversity, but overall fewer differences between the different diets than expected. Our foregut analysis suggests separate and likely important functional differences between colony and wild individuals that were not apparent in the feces. With foreguts dominated by *Lactobacillus*, wild voles may be better suited to survive on a low-quality wild diet [65]. If these bacterial community differences result in beneficial functional differences in digestion, then colony-reared Amargosa voles should be prepared prior to release into the wild to minimize or eliminate those differences to maximize their chance of success. Future work is needed to analyze fecal microbiomes of true wild voles, characterize microbiota from study environments to determine which gut microbes may be transient or resident, and investigate other mechanisms by which captivity and diet may impact the ability of colony-reared animals to survive after release, such as foraging behavior, gut morphology, and digestive enzyme activity.

## Figures and Tables

**Table 1:** Summary of diet treatments provided to study animals.

	Diet Group	*n*	Diet Provided	Duration of Exposure to Diet	Time in Captivity
Sampled for feces	colony	4	Teklad 2018 rodent chow ad libitum	From birth	From birth
	colony-transitional	4	2 pieces Teklad 2018 rodent chow and greenhouse bulrush	24 days	From birth
	wild-captured	4	Native bulrush, slices of carrot, sweet potato, apple, jicama, and Teklad 2018 rodent chow	24–48 h	24–48 h
Sampled for foregut	colony	3	LabDiet 5326 rabbit chow ad libitum	From birth	From birth
	wild	3	Native diet	From birth	n/a

## Supplementary Materials

The following are available online at http://www.mdpi.com/2076-2607/6/3/94/s1, Figure S1: Venn diagrams of the bacterial communities showing the number of shared and unique bacterial taxa in (a) fecal samples and (b) foregut samples. Filtered to exclude taxa present at <0.5%. Table S1: Forage One nutrition analysis of bulrush components collected from a greenhouse (GH) in Davis, CA and from the wild in Tecopa, CA; Table S2: Macronutrient contents of Teklad 2018 rodent chow and LabDiet 5326 rabbit chow; Table S3: ANOVA for phyla-level taxa abundance for fecal samples, Table S4: ANOVA for class-level taxa abundance for fecal samples, Table S5: ANOVA for order-level taxa abundance for fecal samples, Table S6: ANOVA for family-level taxa abundance for fecal samples, Table S7: ANOVA for genus-level taxa abundance for fecal samples, Table S8: PLS-DA genus VIP scores used for loadings name scatterplot of fecal samples, Table S9: Alpha diversity metrics comparing captive, captive-transitional, and wild fecal microbiome samples, Table S10: ANOVA for phyla-level taxa abundance of foregut samples, Table S11: ANOVA for class-level taxa abundance for foregut samples, Table S12: ANOVA for order-level taxa abundance for foregut samples, Table S13: ANOVA for family-level taxa abundance of foregut samples, Table S14: ANOVA for genus-level taxa abundance of foregut samples, Table S15: PLS-DA genus VIP Scores-foregut, Table S16: Alpha diversity metrics comparing captive and wild foregut microbiome samples.
