# Supplementary material for: Conservation Implications of Shifting Gut Microbiomes in Captive-Reared Endangered Voles Intended for Reintroduction into the Wild
Source: Microorganisms. 2018 Sep 12;6(3):94. doi: 10.3390/microorganisms6030094 (PMC6165168; doi:10.3390/microorganisms6030094)
Supplement: Supplementary file 1 [file microorganisms-06-00094-s001.pdf]

Supplementary Materials

Figure S1: Venn diagram of the filtered bacterial communities showing the number of shared and unique core bacterial taxa in (a) fecal samples and (b) foregut. OTU presence was required in all samples within a diet group in order to be retained. Samples were filtered to exclude taxa present at <0.5% abundances.

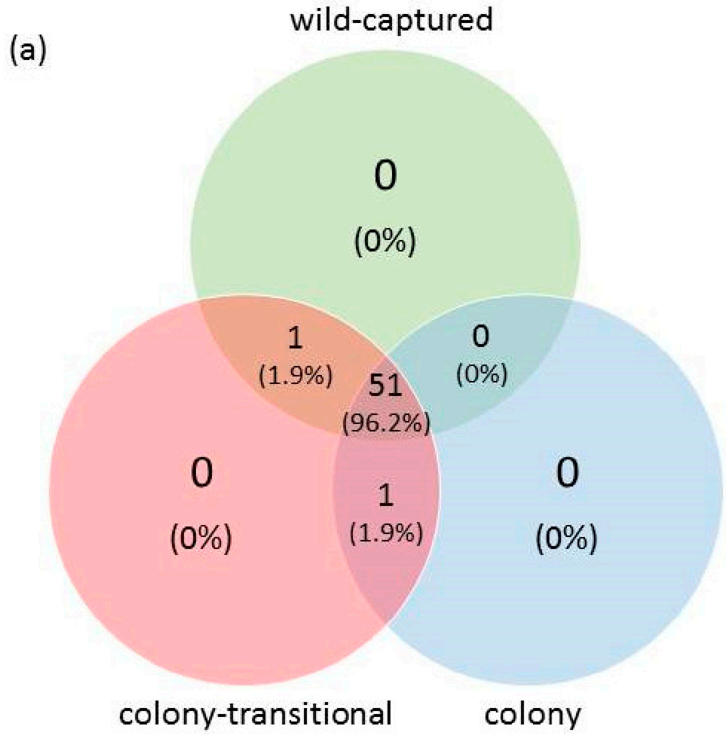

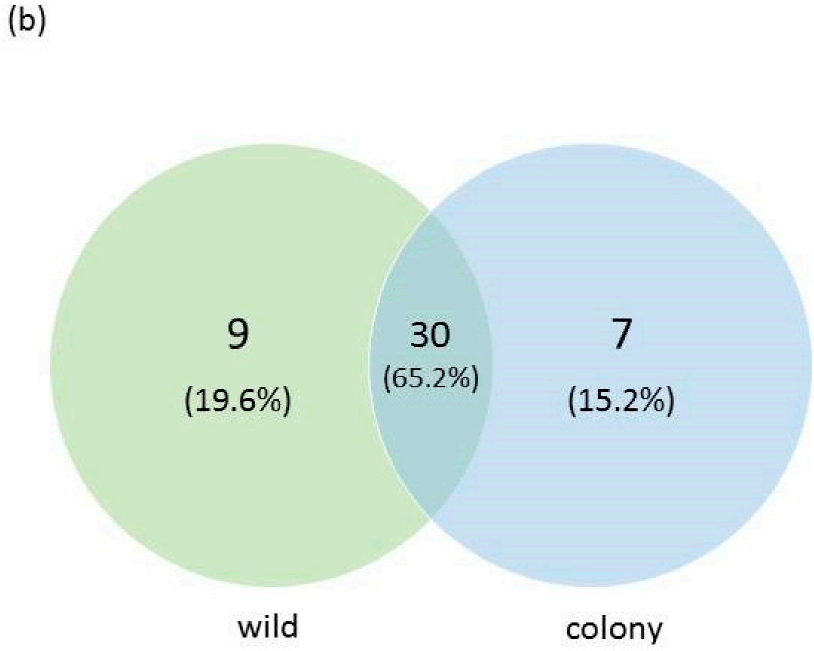

Table S1: Macronutrient analysis of bulrush (Schoenoplectus americanus), components collected from a greenhouse (GH) in Davis, CA and from the wild in Tecopa, CA.

	Leaves		Roots		Rhizomes	
	GH	Wild	GH	Wild	GH	Wild
% moisture	81.9	71.7	91.4	91.9	87.3	88.4
% dry matter	18.1	28.3	8.6	8.1	12.7	11.6
% crude protein	1.8	2.3	0.6	0.6	1.1	0.8
% crude fat	0.5	0.7	0.1	0.1	0.3	0.1
%aNDF	11.7	17.5	7.2	6.5	8.3	5.9
%TDN	10	16	4	4	7	7
ME (kcal/g)	2.02	2	1.71	1.74	1.98	2.06

Table S2: Macronutrient contents of Teklad 2018 rodent chow and LabDiet 5326 rabbit chow.

	Teklad 2018 rodent chow	LabDiet 5326 rabbit chow
% protein	18.6	14.5
% fat (ether extract)	6.2	2.8
% crude fiber	3.5	22.6
% NDF	14.7	42.4
ME (kcal/g)	3.1	1.98

Table S3: Phylum-level Taxa Percent Abundance in Fecal Samples

Phyla-level Taxa	Colony	Colony-transitional	Wild-captured	paired t test	ANOVA p value	ANOVA FDR p value
Bacteroidetes	57.29 ± 7.02[a]	50.67 ± 5.13[a]	52.87 ± 4.32[a]	0.423	0.706	0.8481
Firmicutes	31.4 ± 3.44[a]	40.77 ± 4.47[a]	34.66 ± 4.11[a]	0.166	0.297	0.6534
Spirochaetes	6.29 ± 3.43[a]	4.7 ± 2.54[a]	5.15 ± 2.46[a]	0.761	0.922	0.922
Proteobacteria	4.12 ± 2.04[a]	2.8 ± 0.48[a]	3.95 ± 1.08[a]	0.489	0.763	0.8481
Cyanobacteria	0.63 ± 0.25[a]	0.69 ± 0.38[a]	1.33 ± 0.56[a]	0.928	0.453	0.7119
Actinobacteria	0 ± 0[a]	0 ± 0[a]	0.05 ± 0.02[b]	NA	0.009	0.099
Verrucomicrobia	0 ± 0[a]	0 ± 0[a]	1.03 ± 0.62[a]	NA	0.117	0.3218
Deferribacteres	0 ± 0[a]	0 ± 0[a]	0.11 ± 0.06[a]	NA	0.079	0.3218
Tenericutes	0 ± 0[a]	0 ± 0[a]	0.07 ± 0.04[a]	NA	0.116	0.3218
Elusimicrobia	0.17 ± 0.14[a]	0.23 ± 0.19[a]	0.42 ± 0.36[a]	0.821	0.771	0.8481
Candidatus.Saccharibacter	0 ± 0[a]	0 ± 0[a]	0.31 ± 0.29[a]	NA	0.366	0.671

Values are means ± SEM. [a-b] Means in a row without a common superscript letter differ ($P < 0.05$) as analyzed by one-way ANOVA and the TUKEY test. Paired t-test p value for diet comparison in Colony animals.

Table S4: Class-level Taxa Percent Abundance in Fecal Samples

Class-level Taxa	Colony	Colony-transitional	Wild-captured	paired t test	ANOVA p value	ANOVA FDR p value
Bacteroidia	57.24 ± 7.03^{a}	50.26 ± 5.13^{a}	52.27 ± 4.71^{a}	0.394	0.685	0.7611
Erysipelotrichia	10.78 ± 2.98^{b}	4.9 ± 2.42^{ab}	0.8 ± 0.43^{a}	0.122	0.034	0.34
Clostridia	18.71 ± 5.94^{a}	32.21 ± 4.53^{a}	31.86 ± 4.26^{a}	0.09	0.146	0.4371
Spirochaetia	6.29 ± 3.43^{a}	4.7 ± 2.54^{a}	5.15 ± 2.46^{a}	0.761	0.922	0.922
Bacilli	1.84 ± 0.44^{a}	3.6 ± 1.27^{a}	1.98 ± 0.78^{a}	0.25	0.349	0.6471
Deltaproteobacteria	2.68 ± 2.14^{a}	1.58 ± 0.19^{a}	0.97 ± 0.54^{a}	0.621	0.645	0.7588
Gloeobacteria	0.63 ± 0.25^{a}	0.69 ± 0.38^{a}	1.33 ± 0.56^{a}	0.928	0.453	0.6471
Alphaproteobacteria	1.08 ± 0.89^{a}	0.81 ± 0.27^{a}	2.22 ± 1.22^{a}	0.816	0.515	0.685
Gammaproteobacteria	0.05 ± 0.03^{a}	0.14 ± 0.07^{a}	0.09 ± 0.06^{a}	0.352	0.548	0.685
Epsilonproteobacteria	0.17 ± 0.09^{a}	0.19 ± 0.08^{a}	0.63 ± 0.44^{a}	0.877	0.42	0.6471
Betaproteobacteria	0.11 ± 0.06^{a}	0.04 ± 0.03^{a}	0 ± 0^{a}	0.44	0.194	0.485
Actinobacteria	0 ± 0^{a}	0 ± 0^{a}	0.05 ± 0.02^{b}	NA	0.009	0.18
Sphingobacteriia	0 ± 0^{a}	0.39 ± 0.14^{a}	0.54 ± 0.44^{a}	0.063	0.374	0.6471
Negativicutes	0.06 ± 0.06^{a}	0.06 ± 0.02^{a}	0 ± 0^{a}	0.943	0.424	0.6471
Opitutae	0 ± 0^{a}	0 ± 0^{a}	0.97 ± 0.63^{a}	NA	0.153	0.4371
Deferribacteres	0 ± 0^{a}	0 ± 0^{a}	0.11 ± 0.06^{a}	NA	0.079	0.4371
Mollicutes	0 ± 0^{a}	0 ± 0^{a}	0.07 ± 0.04^{a}	NA	0.116	0.4371
Elusimicrobia	0.17 ± 0.14^{a}	0.23 ± 0.19^{a}	0.42 ± 0.36^{a}	0.821	0.771	0.8116
Candidatus.Saccharibacter	0 ± 0^{a}	0 ± 0^{a}	0.31 ± 0.29^{a}	NA	0.366	0.6471
Verrucomicrobiae	0 ± 0^{a}	0 ± 0^{a}	0.06 ± 0.04^{a}	NA	0.108	0.4371

Values are means ± SEM. $^{a-b}$ Means in a row without a common superscript letter differ ($P < 0.05$) as analyzed by one-way ANOVA and the TUKEY test. Paired t-test p value for diet comparison in Colony animals.

Table S5: Order-level Taxa Percent Abundance in Fecal Samples

Order- level Taxa	Colony	Colony-transitional	Wild-captured	paired t test	ANOVA p value	ANOVA FDR p value
Bacteroidales	57.24 ± 7.03[a]	50.26 ± 5.13[a]	52.27 ± 4.71[a]	0.394	0.685	0.7877
Erysipelotrichales	10.78 ± 2.98[b]	4.9 ± 2.42[ab]	0.8 ± 0.43[a]	0.122	0.034	0.391
Clostridiales	18.7 ± 5.94[a]	32.2 ± 4.54[a]	31.85 ± 4.26[a]	0.09	0.146	0.4571
Spirochaetales	6.29 ± 3.43[a]	4.7 ± 2.54[a]	5.15 ± 2.46[a]	0.761	0.922	0.922
Desulfovibrionales	2.62 ± 2.16[a]	1.34 ± 0.19[a]	0.87 ± 0.56[a]	0.584	0.627	0.759
Lactobacillales	1.69 ± 0.43[a]	3.47 ± 1.27[a]	1.56 ± 0.82[a]	0.251	0.297	0.5736
Gloeobacterales	0.63 ± 0.25[a]	0.69 ± 0.38[a]	1.33 ± 0.56[a]	0.928	0.453	0.5788
Kopriimonadales	0.83 ± 0.75[a]	0.64 ± 0.32[a]	0.45 ± 0.15[a]	0.851	0.852	0.8907
Bacillales	0.15 ± 0.05[ab]	0.13 ± 0.03[a]	0.42 ± 0.11[b]	0.77	0.033	0.391
Campylobacterales	0.17 ± 0.09[a]	0.19 ± 0.08[a]	0.63 ± 0.44[a]	0.877	0.42	0.5736
Burkholderiales	0.11 ± 0.06[a]	0.04 ± 0.03[a]	0 ± 0[a]	0.44	0.194	0.4958
Sphingobacteriales	0 ± 0[a]	0.39 ± 0.14[a]	0.54 ± 0.44[a]	0.063	0.374	0.5736
Enterobacteriales	0 ± 0[a]	0.13 ± 0.06[a]	0.05 ± 0.03[a]	0.125	0.116	0.4571
Rhizobiales	0.17 ± 0.11[a]	0.09 ± 0.06[a]	1.33 ± 1.13[a]	0.611	0.366	0.5736
Selenomonadales	0.06 ± 0.06[a]	0.06 ± 0.02[a]	0 ± 0[a]	0.943	0.424	0.5736
Bdellovibrionales	0.05 ± 0.05[a]	0.24 ± 0.09[a]	0.08 ± 0.04[a]	0.169	0.153	0.4571
Deferribacterales	0 ± 0[a]	0 ± 0[a]	0.11 ± 0.06[a]	NA	0.079	0.4571
Opitutales	0 ± 0[a]	0 ± 0[a]	0.95 ± 0.63[a]	NA	0.159	0.4571
Elusimicrobiales	0.17 ± 0.14[a]	0.23 ± 0.19[a]	0.42 ± 0.36[a]	0.821	0.771	0.8444
Rhodospirillales	0 ± 0[a]	0 ± 0[a]	0.08 ± 0.08[a]	NA	0.405	0.5736
Rhodobacterales	0 ± 0[a]	0 ± 0[a]	0.33 ± 0.29[a]	NA	0.325	0.5736
Candidatus.Saccharibacter	0 ± 0[a]	0 ± 0[a]	0.31 ± 0.29[a]	NA	0.366	0.5736
Verrucomicrobiales	0 ± 0[a]	0 ± 0[a]	0.06 ± 0.04[a]	NA	0.108	0.4571

Values are means ± SEM. [a-b] Means in a row without a common superscript letter differ ($P < 0.05$) as analyzed by one-way ANOVA and the TUKEY test. Paired t-test p value for diet comparison in Colony animals.

Table S6: Family-level Taxa Percent Abundance in Fecal Samples

Family-level Taxa	Colony	Colony-transitional	Wild-captured	paired t test	ANOVA p value	ANOVA FDR p value
Porphyromonadaceae	49.09 ± 7.79[a]	41.94 ± 4.79[a]	39.04 ± 3.13[a]	0.381	0.456	0.559
Erysipelotrichaceae	10.78 ± 2.98[b]	4.9 ± 2.42[ab]	0.8 ± 0.43[a]	0.122	0.034	0.3515
Bacteroidaceae	7.51 ± 1.2[a]	7.01 ± 1.16[a]	7.08 ± 1.33[a]	0.631	0.953	0.953
Eubacteriaceae	2.69 ± 1.08[a]	5.8 ± 1.33[a]	2.25 ± 0.72[a]	0.078	0.087	0.4042
Clostridiaceae	3 ± 0.81[a]	4.14 ± 0.72[a]	11.63 ± 2.97[b]	0.059	0.017	0.3515
Ruminococcaceae	10.96 ± 6.76[a]	14.19 ± 4.55[a]	10.11 ± 2.87[a]	0.51	0.833	0.925
Lachnospiraceae	1.35 ± 0.25[a]	6.69 ± 3.43[a]	4.41 ± 0.89[a]	0.2	0.236	0.5147
Spirochaetaceae	6.29 ± 3.43[a]	4.7 ± 2.54[a]	5.15 ± 2.46[a]	0.761	0.922	0.9469
Rikenellaceae	0.55 ± 0.11[a]	1.23 ± 0.53[a]	2.86 ± 1.11[a]	0.361	0.115	0.4042
Desulfovibrionaceae	2.59 ± 2.13[a]	1.28 ± 0.19[a]	0.82 ± 0.53[a]	0.574	0.613	0.7279
Lactobacillaceae	1.63 ± 0.4[a]	3.4 ± 1.28[a]	1.49 ± 0.8[a]	0.257	0.298	0.5147
Gloeobacterales	0.63 ± 0.25[a]	0.69 ± 0.38[a]	1.33 ± 0.56[a]	0.928	0.453	0.559
Clostridiales	0.54 ± 0.25[a]	1.05 ± 0.24[a]	0.93 ± 0.13[a]	0.034	0.267	0.5147
Kopriimonadaceae	0.83 ± 0.75[a]	0.64 ± 0.32[a]	0.45 ± 0.15[a]	0.851	0.852	0.925
Helicobacteraceae	0.17 ± 0.09[a]	0.19 ± 0.08[a]	0.63 ± 0.44[a]	0.877	0.42	0.559
Prevotellaceae	0.07 ± 0.01[a]	0.06 ± 0[a]	2.55 ± 1.02[b]	0.422	0.023	0.3515
Defluviitaleaceae	0 ± 0[a]	0.03 ± 0.02[a]	0.13 ± 0.09[a]	0.186	0.271	0.5147
Sutterellaceae	0.1 ± 0.06[a]	0 ± 0[a]	0 ± 0[a]	0.18	0.099	0.4042
Bacillaceae	0.08 ± 0.06[a]	0.1 ± 0.03[a]	0.25 ± 0.11[a]	0.861	0.271	0.5147
Peptococcaceae	0 ± 0[a]	0 ± 0[a]	2.31 ± 1.86[a]	NA	0.267	0.5147
Sphingobacteriaceae	0 ± 0[a]	0.39 ± 0.14[a]	0.54 ± 0.44[a]	0.063	0.374	0.559
Marinilabiliaceae	0 ± 0[a]	0 ± 0[a]	0.73 ± 0.56[a]	NA	0.242	0.5147
Enterobacteriaceae	0 ± 0[a]	0.13 ± 0.06[a]	0.05 ± 0.03[a]	0.125	0.116	0.4042
Bacillales	0 ± 0[a]	0 ± 0[a]	0.16 ± 0.07[a]	NA	0.037	0.3515
Christensenellaceae	0 ± 0[a]	0.16 ± 0.13[a]	0 ± 0[a]	0.316	0.286	0.5147
Veillonellaceae	0.06 ± 0.06[a]	0.06 ± 0.02[a]	0 ± 0[a]	0.943	0.424	0.559
Rhizobiaceae	0.16 ± 0.11[a]	0.09 ± 0.06[a]	1.18 ± 1.1[a]	0.62	0.44	0.559
Streptococcaceae	0.04 ± 0.04[a]	0.06 ± 0.03[a]	0.06 ± 0.03[a]	0.431	0.891	0.9405
Bdellovibrionaceae	0.05 ± 0.05[a]	0.24 ± 0.09[a]	0.08 ± 0.04[a]	0.169	0.153	0.4316
Deferribacteraceae	0 ± 0[a]	0 ± 0[a]	0.11 ± 0.06[a]	NA	0.079	0.4042
Desulfomicrobiaceae	0 ± 0[a]	0.05 ± 0.03[a]	0 ± 0[a]	0.196	0.117	0.4042
Opitutaceae	0 ± 0[a]	0 ± 0[a]	0.95 ± 0.63[a]	NA	0.159	0.4316
Rhodospirillaceae	0 ± 0[a]	0 ± 0[a]	0.08 ± 0.08[a]	NA	0.405	0.559
Elusimicrobiaceae	0.17 ± 0.14[a]	0.23 ± 0.19[a]	0.42 ± 0.36[a]	0.821	0.771	0.8878
Hyphomonadaceae	0 ± 0[a]	0 ± 0[a]	0.33 ± 0.29[a]	NA	0.325	0.537
Candidatus.Saccharibacter	0 ± 0[a]	0 ± 0[a]	0.31 ± 0.29[a]	NA	0.366	0.559
Brucellaceae	0 ± 0[a]	0 ± 0[a]	0.15 ± 0.1[a]	NA	0.148	0.4316
Verrucomicrobiaceae	0 ± 0[a]	0 ± 0[a]	0.06 ± 0.04[a]	NA	0.108	0.4042

Values are means ± SEM. [a-b] Means in a row without a common superscript letter differ ($P < 0.05$) as analyzed by one-way ANOVA and the TUKEY test. Paired t-test p value for diet comparison in Colony animals.

Table S7: Genus-level Taxa Percent Abundance in Fecal Samples

Genus-level Taxa	Colony	Colony-transitional	Wild-captured	paired t test	ANOVA p value	ANOVA FDR p value
Barnesiella	41.67 ± 7.37[a]	35.54 ± 4.93[a]	31.49 ± 3.61[a]	0.38	0.456	0.57
Allobaculum	10.63 ± 3[b]	4.7 ± 2.38[ab]	0.26 ± 0.09[a]	0.119	0.027	0.3853
Bacteroides	7.51 ± 1.2[a]	7.01 ± 1.16[a]	7.08 ± 1.33[a]	0.631	0.953	0.953
Eubacterium	2.68 ± 1.08[a]	5.71 ± 1.3[a]	2.24 ± 0.73[a]	0.081	0.091	0.3978
Tannerella	3.58 ± 0.56[a]	3.29 ± 0.35[a]	4.78 ± 0.62[a]	0.754	0.158	0.4434
Paludibacter	2.68 ± 0.52[b]	1.71 ± 0.19[ab]	0.94 ± 0.46[a]	0.163	0.046	0.3853
Clostridium	2.99 ± 0.81[a]	4.13 ± 0.72[a]	11.6 ± 2.96[b]	0.06	0.017	0.3853
Porphyromonas	1 ± 0.16[a]	1.27 ± 0.23[a]	1.48 ± 0.35[a]	0.459	0.459	0.57
Treponema	6.01 ± 3.5[a]	4.22 ± 2.58[a]	4.76 ± 2.41[a]	0.732	0.903	0.9165
Ruminococcus	9.16 ± 6.8[a]	11.25 ± 5.06[a]	6.66 ± 3.13[a]	0.67	0.827	0.8756
Anaerostipes	0.35 ± 0.13[a]	0.15 ± 0.04[a]	0.59 ± 0.17[a]	0.215	0.096	0.3978
Alistipes	0.51 ± 0.12[a]	1.15 ± 0.49[a]	2.55 ± 1.24[a]	0.357	0.215	0.544
Desulfovibrio	2.57 ± 2.12[a]	1.25 ± 0.18[a]	0.8 ± 0.51[a]	0.571	0.606	0.6902
Lactobacillus	1.63 ± 0.4[a]	3.39 ± 1.28[a]	1.49 ± 0.8[a]	0.257	0.299	0.57
Gloeobacter	0.63 ± 0.25[a]	0.69 ± 0.38[a]	1.33 ± 0.56[a]	0.928	0.453	0.57
Oscillospira	0.67 ± 0.22[a]	1.01 ± 0.35[a]	1.34 ± 0.48[a]	0.176	0.471	0.5719
Spirochaeta	0.27 ± 0.09[a]	0.49 ± 0.21[a]	0.39 ± 0.08[a]	0.241	0.581	0.6812
Lachnoclostridium	0.5 ± 0.13[a]	4.49 ± 3.65[a]	1.08 ± 0.23[a]	0.339	0.392	0.57
Roseburia	0.12 ± 0.05[a]	0.69 ± 0.51[a]	0.66 ± 0.32[a]	0.344	0.461	0.57
Robinsoniella	0.26 ± 0.06[a]	0.44 ± 0.1[a]	1.58 ± 0.59[a]	0.296	0.051	0.3853
Ruminiclostridium	0.53 ± 0.27[a]	1.36 ± 0.73[a]	1.21 ± 0.71[a]	0.185	0.609	0.6902
Kopriimonas	0.83 ± 0.75[a]	0.64 ± 0.32[a]	0.45 ± 0.15[a]	0.851	0.852	0.8778
Acetivibrio	0.08 ± 0.03[a]	0.13 ± 0.02[a]	0.13 ± 0.09[a]	0.406	0.788	0.8505
Pseudoflavonifractor	0.45 ± 0.22[a]	0.84 ± 0.19[a]	0.24 ± 0.07[a]	0.064	0.092	0.3978
Helicobacter	0.17 ± 0.09[a]	0.19 ± 0.08[a]	0.63 ± 0.44[a]	0.877	0.42	0.57
Coprobacter	0.08 ± 0.01[a]	0.05 ± 0.02[a]	0.09 ± 0.06[a]	0.166	0.702	0.7826
Papillibacter	0.11 ± 0.03[a]	0.15 ± 0.06[a]	0.07 ± 0.04[a]	0.37	0.417	0.57
Candidatus.Soleaferrea	0.23 ± 0.19[a]	0.1 ± 0.01[a]	0.35 ± 0.12[a]	0.541	0.443	0.57
Vallitalea	0 ± 0[a]	0.03 ± 0.02[a]	0.13 ± 0.09[a]	0.186	0.271	0.57
Sutterella	0.1 ± 0.06[a]	0 ± 0[a]	0 ± 0[a]	0.18	0.099	0.3978
Blautia	0.03 ± 0.02[a]	0.71 ± 0.53[a]	0.17 ± 0.03[a]	0.3	0.305	0.57
Prevotella	0 ± 0[a]	0 ± 0[a]	1.34 ± 0.61[a]	NA	0.039	0.3853
Bacillus	0.06 ± 0.06[a]	0.06 ± 0.04[a]	0.24 ± 0.11[a]	0.994	0.216	0.544
Turicibacter	0.13 ± 0.05[a]	0.18 ± 0.05[a]	0.51 ± 0.4[a]	0.245	0.491	0.5858
Anaerophaga	0 ± 0[a]	0 ± 0[a]	0.73 ± 0.56[a]	NA	0.242	0.5674
Butyricimonas	0 ± 0[a]	0 ± 0[a]	0.06 ± 0.04[a]	NA	0.163	0.4434
Dehalobacterium	0 ± 0[a]	0 ± 0[a]	0.16 ± 0.07[a]	NA	0.037	0.3853
Selenomonas	0.06 ± 0.06[a]	0.06 ± 0.02[a]	0 ± 0[a]	0.943	0.424	0.57

Genus-level Taxa	Colony	Colony-transitional	Wild-captured	paired t test	ANOVA p value	ANOVA FDR p value
Intestinimonas	0 ± 0[a]	0.07 ± 0.03[a]	0.17 ± 0.09[a]	0.094	0.147	0.4434
Christensenella	0 ± 0[a]	0.16 ± 0.13[a]	0 ± 0[a]	0.316	0.286	0.57
Citrobacter	0 ± 0[a]	0.1 ± 0.07[a]	0.05 ± 0.03[a]	0.232	0.289	0.57
Rikenella	0 ± 0[a]	0.06 ± 0.04[a]	0.3 ± 0.16[a]	0.234	0.116	0.3978
Subdoligranulum	0 ± 0[a]	0.04 ± 0.02[a]	0.08 ± 0.06[a]	0.197	0.306	0.57
Kaistia	0.16 ± 0.11[a]	0.09 ± 0.06[a]	1.18 ± 1.1[a]	0.62	0.44	0.57
Lactococcus	0.03 ± 0.03[a]	0.05 ± 0.03[a]	0 ± 0[a]	0.382	0.398	0.57
Pedobacter	0 ± 0[a]	0.05 ± 0.05[a]	0.49 ± 0.42[a]	0.391	0.349	0.57
Natranaerovirga	0 ± 0[a]	0.07 ± 0.04[ab]	0.32 ± 0.12[b]	0.184	0.03	0.3853
Sphingobacterium	0 ± 0[a]	0.33 ± 0.15[a]	0 ± 0[a]	0.119	0.04	0.3853
Acetobacterium	0 ± 0[a]	0.09 ± 0.05[a]	0 ± 0[a]	0.183	0.102	0.3978
Hallella	0 ± 0[a]	0 ± 0[a]	0.52 ± 0.18[b]	NA	0.008	0.3853
Desulfotomaculum	0 ± 0[a]	0 ± 0[a]	2.27 ± 1.86[a]	NA	0.278	0.57
Vampirovibrio	0.05 ± 0.05[a]	0.24 ± 0.09[a]	0.08 ± 0.04[a]	0.169	0.153	0.4434
Sporobacter	0 ± 0[a]	0 ± 0[a]	0.04 ± 0.04[a]	NA	0.405	0.57
Coprococcus	0 ± 0[a]	0.11 ± 0.1[a]	0.05 ± 0.03[a]	0.315	0.407	0.57
Mucispirillum	0 ± 0[a]	0 ± 0[a]	0.11 ± 0.06[a]	NA	0.079	0.3978
Desulfomicrobium	0 ± 0[a]	0.05 ± 0.03[a]	0 ± 0[a]	0.196	0.117	0.3978
Paraprevotella	0 ± 0[a]	0 ± 0[a]	0.69 ± 0.37[a]	NA	0.076	0.3978
Opitutus	0 ± 0[a]	0 ± 0[a]	0.95 ± 0.63[a]	NA	0.159	0.4434
Faecalibacterium	0.07 ± 0.07[a]	0.05 ± 0.05[a]	0.1 ± 0.05[a]	0.391	0.837	0.8756
Odoribacter	0 ± 0[a]	0 ± 0[a]	0.15 ± 0.12[a]	NA	0.238	0.5674
Flavonifractor	0 ± 0[a]	0 ± 0[a]	0.2 ± 0.1[a]	NA	0.068	0.3978
Butyrivibrio	0 ± 0[a]	0 ± 0[a]	0.03 ± 0.03[a]	NA	0.405	0.57
Marispirillum	0 ± 0[a]	0 ± 0[a]	0.08 ± 0.08[a]	NA	0.405	0.57
Elusimicrobium	0.17 ± 0.14[a]	0.23 ± 0.19[a]	0.42 ± 0.36[a]	0.821	0.771	0.8456
Candidatus.Saccharimonas	0 ± 0[a]	0 ± 0[a]	0.31 ± 0.29[a]	NA	0.366	0.57
Hyphomonas	0 ± 0[a]	0 ± 0[a]	0.33 ± 0.29[a]	NA	0.325	0.57
Akkermansia	0 ± 0[a]	0 ± 0[a]	0.06 ± 0.04[a]	NA	0.107	0.3978
Ochrobactrum	0 ± 0[a]	0 ± 0[a]	0.1 ± 0.1[a]	NA	0.405	0.57

Values are means ± SEM. [a-b] Means in a row without a common superscript letter differ ($P < 0.05$) as analyzed by one-way ANOVA and the TUKEY test. Paired t-test p value for diet comparison in Colony animals.

Table S8: PLS-DA Genus VIP scores used for loadings name scatterplot of fecal samples

Bacteria	VIP
Erysipelothrix	5.205
Sphingobacterium	4.415
Acetobacterium	4.163
Azospirillum	3.818
Vampirovibrio	3.614
Eubacterium	3.517
Staphylococcus	3.398
Lactobacillus	3.094
Oxalobacter	2.976
Plasticicumulans	2.949
Tyzzerella	2.942
Pseudobutyrivibrio	2.909
Marvinbryantia	2.787
Citrobacter	2.626
Facklamia	2.592
Blautia	2.493
Pseudoflavonifractor	2.478
Paraeggerthella	2.256
Coprococcus	2.251
Candidatus.Nucleicultrix	2.194
Christensenella	2.186
Allobaculum	2.186
Lachnoclostridium	2.116
Methylobacterium	2.095
Curtobacterium	2.061
Psychrobacter	2.002

Table S9. Alpha diversity metrics comparing colony, colony-transitional, and wild-captured fecal microbiome samples

	Colony		Colony-transitional		Wild-captured		Tukey's post-hoc pairwise comparisons			Bonferroni corrected p-value		
	mean	sd	mean	sd	mean	sd	Colony vs Colony-transitional	Colony vs Wild-captured	Colony-transitional vs Wild-captured	Colony vs Colony-transitional	Colony vs Wild-captured	Colony-transitional vs Wild-captured
Chao	2118.650	50.755	2170.212	65.005	2104.924	101.606	1.083	-0.209	-0.937	1.000	1.000	1.000
Faith's PD	77.023	2.314	79.639	2.387	82.446	2.404	1.363	2.815	1.435	0.684	0.168	0.657
Goods Coverage	0.989	0.000	0.989	0.000	0.990	0.001	-0.132	2.432	2.396	1.000	0.093	0.189
Observed Species	1546.450	57.835	1617.650	62.644	1639.075	73.062	1.446	1.722	0.386	0.837	0.552	1.000
Shannon	6.568	0.237	6.768	0.405	7.719	0.305	0.740	5.161	3.249	1.000	0.075	0.111
Simpson's	0.947	0.016	0.951	0.027	0.983	0.005	0.217	3.866	2.075	1.000	0.072	0.174

Table S10: Phylum-level Taxa Percent Abundance in Foregut Samples

Phyla-level Taxa	Colony	Wild	P value	FDR pvalue
Unassigned	0 ± 0[a]	0.19 ± 0.19[a]	0.374	0.588
Euryarchaeota	0 ± 0[a]	0.25 ± 0.17[a]	0.216	0.588
Actinobacteria	0.6 ± 0.47[a]	2.2 ± 1.72[a]	0.42	0.588
Bacteroidetes	8.38 ± 5.69[a]	13.86 ± 13.56[a]	0.728	0.784
Cyanobacteria	3.28 ± 2.21[a]	13.06 ± 8.43[a]	0.324	0.588
Deferribacteres	0 ± 0[a]	0.11 ± 0.11[a]	0.374	0.588
Firmicutes	58.83 ± [illegible]	64.08 ± 11.38[a]	0.859	0.859
Fusobacteria	3.13 ± 3.11[a]	0.15 ± 0.09[a]	0.391	0.588
Proteobacteria	25.13 ± [illegible]	4.82 ± 0.9[a]	0.372	0.588
Spirochaetes	0 ± 0[a]	0.85 ± 0.85[a]	0.374	0.588
Tenericutes	0.22 ± 0.22[a]	0.06 ± 0.03[a]	0.521	0.6631
Verrucomicrobia	0 ± 0[a]	0.08 ± 0.08[a]	0.374	0.588
Chromerida	0.09 ± 0.09[a]	0 ± 0[a]	0.374	0.588
Ascomycota	0.1 ± 0.1[a]	0.04 ± 0.04[a]	0.595	0.6942

Values are means ± SEM. [a-b] Means in a row without a common superscript letter differ ($P < 0.05$) as analyzed by one-way ANOVA and the TUKEY post-hoc test.

Table S11: Class-level Taxa Percent Abundance in Foregut Samples

Class-level Taxa	Colony	Wild	p value	FDR pvalue
Unassigned	0 ± 0[a]	0.19 ± 0.19[a]	0.374	0.5163
Methanobacteria	0 ± 0[a]	0.19 ± 0.19[a]	0.374	0.5163
Actinobacteria	0.6 ± 0.47[a]	2.19 ± 1.72[a]	0.423	0.5163
Bacteroidia	8.27 ± 5.68[a]	12.95 ± 12.71[a]	0.753	0.7872
Cytophagia	0 ± 0[a]	0.47 ± 0.47[a]	0.374	0.5163
Sphingobacteriia	0.03 ± 0.03[a]	0.4 ± 0.4[a]	0.41	0.5163
Cyanobacteria	3.19 ± 2.23[a]	13.01 ± 8.46[a]	0.324	0.5163
Gloeobacteria	0.09 ± 0.09[a]	0 ± 0[a]	0.374	0.5163
Deferribacteres	0 ± 0[a]	0.11 ± 0.11[a]	0.374	0.5163
Bacilli	3.44 ± 2.74[a]	57.05 ± 16.48[b]	0.033	0.5163
Clostridia	6.56 ± 3.19[a]	6.54 ± 6.37[a]	0.998	0.998
Erysipelotrichia	48.78 ± 25.08[a]	0.45 ± 0.15[a]	0.126	0.5163
Fusobacteriia	3.13 ± 3.11[a]	0.15 ± 0.09[a]	0.391	0.5163
Alphaproteobacteria	0.16 ± 0.08[a]	0.63 ± 0.31[a]	0.207	0.5163
Betaproteobacteria	0.83 ± 0.77[a]	0.18 ± 0.05[a]	0.449	0.5163
Deltaproteobacteria	2.76 ± 1.67[a]	0.71 ± 0.62[a]	0.314	0.5163
Epsilonproteobacteria	0 ± 0[a]	0.12 ± 0.12[a]	0.374	0.5163
Gammaproteobacteria	21.38 ± 20.66[a]	3.18 ± 1.53[a]	0.429	0.5163
Spirochaetia	0 ± 0[a]	0.85 ± 0.85[a]	0.374	0.5163
Mollicutes	0.22 ± 0.22[a]	0.06 ± 0.03[a]	0.521	0.5706
Verrucomicrobiae	0 ± 0[a]	0.08 ± 0.08[a]	0.374	0.5163
Chromerida	0.09 ± 0.09[a]	0 ± 0[a]	0.374	0.5163
Dothideomycetes	0.09 ± 0.09[a]	0 ± 0[a]	0.374	0.5163

Values are means ± SEM. [a-b] Means in a row without a common superscript letter differ ($P < 0.05$) as analyzed by one-way ANOVA and the TUKEY post-hoc test.

Table S12: Order-level Taxa Percent Abundance in Foregut Samples

Order- level Taxa	Colony	Wild	p value	FDR pvalue
Unassigned	0 ± 0[a]	0.19 ± 0.19[a]	0.374	0.503
Methanobacteriales	0 ± 0[a]	0.19 ± 0.19[a]	0.374	0.503
Actinomycetales	0.6 ± 0.47[a]	1.98 ± 1.81[a]	0.501	0.5615
Coriobacteriales	0 ± 0[a]	0.18 ± 0.18[a]	0.374	0.503
Bacteroidales	8.27 ± 5.68[a]	12.95 ± 12.71[a]	0.753	0.7758
Cytophagales	0 ± 0[a]	0.47 ± 0.47[a]	0.374	0.503
Sphingobacteriales	0.03 ± 0.03[a]	0.4 ± 0.4[a]	0.41	0.503
Cillatoriales	3.17 ± 2.24[a]	12.96 ± 8.48[a]	0.327	0.503
Gloeobacterales	0.09 ± 0.09[a]	0 ± 0[a]	0.374	0.503
Deferribacterales	0 ± 0[a]	0.11 ± 0.11[a]	0.374	0.503
Bacillales	0.35 ± 0.22[a]	0.86 ± 0.53[a]	0.426	0.503
Lactobacillales	3.09 ± 2.53[a]	56.2 ± 16.43[b]	0.033	0.503
Clostridiales	6.56 ± 3.19[a]	6.54 ± 6.37[a]	0.998	0.998
Erysipelotrichales	48.78 ± 25.08[a]	0.45 ± 0.15[a]	0.126	0.503
Fusobacteriales	3.13 ± 3.11[a]	0.15 ± 0.09[a]	0.391	0.503
Rhizobiales	0.04 ± 0.04[a]	0.17 ± 0.13[a]	0.373	0.503
Rhodobacterales	0 ± 0[a]	0.25 ± 0.21[a]	0.3	0.503
Rhodospirillales	0 ± 0[a]	0.06 ± 0.06[a]	0.374	0.503
Sphingomonadales	0 ± 0[a]	0.05 ± 0.02[a]	0.117	0.503
Burkholderiales	0.26 ± 0.23[a]	0.09 ± 0.06[a]	0.512	0.5615
Neisseriales	0.55 ± 0.55[a]	0.06 ± 0.06[a]	0.429	0.503
Desulfovibrionales	2.75 ± 1.67[a]	0.65 ± 0.62[a]	0.304	0.503
Campylobacterales	0 ± 0[a]	0.12 ± 0.12[a]	0.374	0.503
Enterobacteriales	0.44 ± 0.44[a]	0 ± 0[a]	0.374	0.503
Eanospirillales	0 ± 0[a]	0.07 ± 0.07[a]	0.374	0.503
Pasteurellales	19.7 ± 19.13[a]	2.79 ± 1.47[a]	0.428	0.503
Pseudomonadales	1.07 ± 0.95[a]	0.1 ± 0.07[a]	0.371	0.503
Vibrionales	0 ± 0[a]	0.09 ± 0.09[a]	0.374	0.503
Spirochaetales	0 ± 0[a]	0.85 ± 0.85[a]	0.374	0.503
Acholeplasmatales	0.17 ± 0.17[a]	0.06 ± 0.03[a]	0.563	0.5982
Mycoplasmatales	0.05 ± 0.05[a]	0 ± 0[a]	0.374	0.503
Verrucomicrobiales	0 ± 0[a]	0.08 ± 0.08[a]	0.374	0.503
Chromerida	0.09 ± 0.09[a]	0 ± 0[a]	0.374	0.503
Pleosporales	0.09 ± 0.09[a]	0 ± 0[a]	0.374	0.503

Values are means ± SEM. [a-b] Means in a row without a common superscript letter differ ($P < 0.05$) as analyzed by one-way ANOVA and the TUKEY post-hoc test.

Table S13: Family-level Taxa Percent Abundance in Foregut Samples

Family-level Taxa	Colony	Wild	p value	FDR pvalue
Unassigned	0 ± 0[a]	0.19 ± 0.19[a]	0.374	0.5747
Methanobacteriaceae	0 ± 0[a]	0.19 ± 0.19[a]	0.374	0.5747
Corynebacteriaceae	0.23 ± 0.19[a]	1.62 ± 1.62[a]	0.443	0.5747
Micrococcaceae	0.18 ± 0.11[a]	0.08 ± 0.05[a]	0.463	0.5814
Coriobacteriaceae	0 ± 0[a]	0.18 ± 0.18[a]	0.374	0.5747
Bacteroidaceae	1.67 ± 1.33[a]	0.91 ± 0.89[a]	0.661	0.6999
Porphyromonadaceae	6.41 ± 5.94[a]	10.6 ± 10.44[a]	0.744	0.7726
Prevotellaceae	0 ± 0[a]	1.2 ± 1.2[a]	0.374	0.5747
Rikenellacee	0 ± 0[a]	0.13 ± 0.13[a]	0.374	0.5747
Cytophagaceae	0 ± 0[a]	0.47 ± 0.47[a]	0.374	0.5747
Sphingobacteriaceae	0 ± 0[a]	0.4 ± 0.4[a]	0.374	0.5747
Oscillatoriales	3.04 ± 2.11[a]	11.95 ± 7.79[a]	0.331	0.5747
Gloeobacterales	0.09 ± 0.09[a]	0 ± 0[a]	0.374	0.5747
Deferribacteraceae	0 ± 0[a]	0.11 ± 0.11[a]	0.374	0.5747
Bacillaceae	0 ± 0[a]	0.09 ± 0.07[a]	0.233	0.5747
Bacillales	0.09 ± 0.09[a]	0.11 ± 0.02[a]	0.853	0.853
Staphylococcaceae	0.18 ± 0.12[a]	0.55 ± 0.55[a]	0.545	0.6238
Aerococcaceae	0.13 ± 0.13[a]	0 ± 0[a]	0.374	0.5747
Enterococcaceae	0.06 ± 0.06[a]	0 ± 0[a]	0.374	0.5747
Lactobacillaceae	0.38 ± 0.08[a]	42.24 ± 16.44[a]	0.064	0.5747
Streptococcaceae	2.34 ± 2.25[a]	9.81 ± 8.59[a]	0.447	0.5747
Christensenellaceae	3.77 ± 1.91[a]	0 ± 0[a]	0.12	0.5747
Clostridiaceae	0.59 ± 0.31[a]	1.91 ± 1.87[a]	0.526	0.6238
Clostridiales	0.04 ± 0.04[a]	0.64 ± 0.64[a]	0.404	0.5747
Eubacteriaceae	0.23 ± 0.13[a]	0.67 ± 0.67[a]	0.555	0.6238
Gracilibacteraceae	0.56 ± 0.28[a]	0 ± 0[a]	0.116	0.5747
Lachnospiraceae	0.58 ± 0.3[a]	1.27 ± 1.27[a]	0.625	0.675
Ruminococcaceae	0.68 ± 0.34[a]	1.85 ± 1.85[a]	0.566	0.6238
Erysipelotrichaceae	48.6 ± 25.03[a]	0.43 ± 0.15[a]	0.127	0.5747
Sobacteriales.Other	0 ± 0[a]	0.04 ± 0.04[a]	0.374	0.5747
Sobacteriaceae	2.52 ± 2.52[a]	0.08 ± 0.04[a]	0.387	0.5747
Leptotrichiaceae	0.39 ± 0.39[a]	0 ± 0[a]	0.374	0.5747
Rhodobacteraceae	0 ± 0[a]	0.22 ± 0.19[a]	0.308	0.5747
Rhodospirillaceae	0 ± 0[a]	0.06 ± 0.06[a]	0.374	0.5747
Alcaligenaceae	0.1 ± 0.1[a]	0 ± 0[a]	0.374	0.5747
Comamonadaceae	0.11 ± 0.11[a]	0 ± 0[a]	0.374	0.5747
Oxalobacteraceae	0 ± 0[a]	0.06 ± 0.06[a]	0.374	0.5747
Neisseriaceae	0.52 ± 0.52[a]	0.06 ± 0.06[a]	0.428	0.5747
Desulfovibrionaceae	2.73 ± 1.67[a]	0.58 ± 0.58[a]	0.291	0.5747

Family-level Taxa	Colony	Wild	p value	FDR pvalue
Helicobacteraceae	0 ± 0[a]	0.12 ± 0.12[a]	0.374	0.5498
Enterobacteriaceae	0.44 ± 0.44[a]	0 ± 0[a]	0.374	0.5498
Halomonadaceae	0 ± 0[a]	0.07 ± 0.07[a]	0.374	0.5498
Pasteurellaceae	19.7 ± 19.13[a]	2.79 ± 1.47[a]	0.428	0.5498
Moraxellaceae	0.69 ± 0.69[a]	0.08 ± 0.06[a]	0.432	0.5498
Pseudomonadaceae	0.37 ± 0.27[a]	0 ± 0[a]	0.242	0.5498
Vibrionaceae	0 ± 0[a]	0.09 ± 0.09[a]	0.374	0.5498
Spirochaetaceae	0 ± 0[a]	0.85 ± 0.85[a]	0.374	0.5498
Acholeplasmataceae	0.17 ± 0.17[a]	0.06 ± 0.03[a]	0.563	0.6142
Mycoplasmataceae	0.05 ± 0.05[a]	0 ± 0[a]	0.374	0.5498
Verrucomicrobiaceae	0 ± 0[a]	0.08 ± 0.08[a]	0.374	0.5498
Chromerida	0.09 ± 0.09[a]	0 ± 0[a]	0.374	0.5498

Values are means ± SEM. [a-b] Means in a row without a common superscript letter differ ($P < 0.05$) as analyzed by one-way ANOVA and the TUKEY post-hoc test.

Table S14: Genus-level Taxa Percent Abundance in Foregut Samples

Genus-level Taxa	Colony	Wild	p value	FDR pvalue
Unassigned	0 ± 0[a]	0.19 ± 0.19[a]	0.374	0.5538
Methanobrevibacter	0 ± 0[a]	0.19 ± 0.19[a]	0.374	0.5538
Corynebacterium	0.24 ± 0.2[a]	1.78 ± 1.75[a]	0.433	0.5917
Rothia	0 ± 0[a]	0.05 ± 0.05[a]	0.374	0.5538
Yaniella	0.08 ± 0.04[a]	0 ± 0[a]	0.118	0.5538
Enterorhabdus	0 ± 0[a]	0.05 ± 0.05[a]	0.374	0.5538
Bacteroides	1.74 ± 1.4[a]	0.92 ± 0.89[a]	0.646	0.7106
Barnesiella	5.91 ± 5.73[a]	9.33 ± 9.17[a]	0.767	0.809
Dysgonomonas	0.08 ± 0.08[a]	0 ± 0[a]	0.374	0.5538
Paludibacter	0 ± 0[a]	0.13 ± 0.13[a]	0.374	0.5538
Porphyromonas	0.1 ± 0.1[a]	0.27 ± 0.27[a]	0.59	0.6681
Tannerella	0.29 ± 0.29[a]	0.91 ± 0.91[a]	0.555	0.6493
Prevotella	0 ± 0[a]	1.15 ± 1.15[a]	0.374	0.5538
Alistipes	0 ± 0[a]	0.07 ± 0.07[a]	0.374	0.5538
Rikenella	0 ± 0[a]	0.07 ± 0.07[a]	0.374	0.5538
Adhaeribacter	0 ± 0[a]	0.38 ± 0.38[a]	0.374	0.5538
Cytophaga	0 ± 0[a]	0.08 ± 0.08[a]	0.374	0.5538
Pedobacter	0 ± 0[a]	0.39 ± 0.39[a]	0.374	0.5538
Halospirulina	3.17 ± 2.24[a]	12.96 ± 8.48[a]	0.327	0.5538
Oeobacter	0.09 ± 0.09[a]	0 ± 0[a]	0.374	0.5538
Mucispirillum	0 ± 0[a]	0.11 ± 0.11[a]	0.374	0.5538
Bacillus	0 ± 0[a]	0.08 ± 0.08[a]	0.374	0.5538
Dehalobacterium	0 ± 0[a]	0.05 ± 0.05[a]	0.374	0.5538
Mella	0.1 ± 0.1[a]	0.06 ± 0.03[a]	0.767	0.809
Salinicoccus	0.04 ± 0.04[a]	0 ± 0[a]	0.374	0.5538
Staphylococcus	0.11 ± 0.11[a]	0.58 ± 0.58[a]	0.473	0.607
Aerococcus	0.13 ± 0.13[a]	0 ± 0[a]	0.374	0.5538
Vagococcus	0.06 ± 0.06[a]	0 ± 0[a]	0.374	0.5538
Lactobacillus	0.38 ± 0.07[a]	45.51 ± 18.57[a]	0.072	0.5538
Lactococcus	0.08 ± 0.08[a]	0.09 ± 0.09[a]	0.947	0.9595
Streptococcus	2.38 ± 2.29[a]	10.57 ± 9.24[a]	0.438	0.5917
Christensenella	3.79 ± 1.92[a]	0 ± 0[a]	0.12	0.5538
Clostridium	0.55 ± 0.3[a]	1.91 ± 1.88[a]	0.514	0.6488
Flavonifractor	0 ± 0[a]	0.18 ± 0.18[a]	0.374	0.5538
Intestinimonas	0 ± 0[a]	0.06 ± 0.06[a]	0.374	0.5538
Pseudoflavonifractor	0 ± 0[a]	0.41 ± 0.41[a]	0.374	0.5538
Eubacterium	0.23 ± 0.13[a]	0.65 ± 0.65[a]	0.562	0.6493
Acilibacter	0.56 ± 0.28[a]	0 ± 0[a]	0.116	0.5538
Blautia	0.04 ± 0.04[a]	0.12 ± 0.12[a]	0.562	0.6493

Genus-level Taxa	Colony	Wild	p value	FDR pvalue
Dorea	0.1 ± 0.1[a]	0.14 ± 0.14[a]	0.841	0.8751
Lachnoclostridium	0.16 ± 0.09[a]	0.09 ± 0.09[a]	0.616	0.6874
Robinsoniella	0.05 ± 0.05[a]	0.77 ± 0.77[a]	0.405	0.5698
Roseburia	0.09 ± 0.09[a]	0.08 ± 0.08[a]	0.992	0.992
Acetivibrio	0 ± 0[a]	0.06 ± 0.06[a]	0.374	0.5538
Oscillospira	0.03 ± 0.03[a]	0.21 ± 0.21[a]	0.456	0.599
Ruminiclostridium	0.12 ± 0.12[a]	0.36 ± 0.36[a]	0.565	0.6493
Ruminococcus	0.34 ± 0.2[a]	1.08 ± 1.08[a]	0.537	0.6493
Sporobacter	0.13 ± 0.07[a]	0.08 ± 0.08[a]	0.674	0.731
Allobaculum	48.76 ± 25.08[a]	0.32 ± 0.04[a]	0.126	0.5538
Turicibacter	0 ± 0[a]	0.11 ± 0.11[a]	0.374	0.5538
Fusobacteriales.Other.Other	0 ± 0[a]	0.05 ± 0.05[a]	0.374	0.5538
Fusobacterium	2.66 ± 2.66[a]	0.08 ± 0.05[a]	0.388	0.5637
Leptotrichia	0.41 ± 0.41[a]	0 ± 0[a]	0.374	0.5538
Paracoccus	0 ± 0[a]	0.05 ± 0.05[a]	0.374	0.5538
Ruegeria	0 ± 0[a]	0.06 ± 0.06[a]	0.374	0.5538
Azospirillum	0 ± 0[a]	0.06 ± 0.06[a]	0.374	0.5538
Alcaligenes	0.1 ± 0.1[a]	0 ± 0[a]	0.374	0.5538
Comamonas	0.08 ± 0.08[a]	0 ± 0[a]	0.374	0.5538
Oxalobacter	0 ± 0[a]	0.06 ± 0.06[a]	0.374	0.5538
Kingella	0.37 ± 0.37[a]	0.06 ± 0.06[a]	0.459	0.599
Stenoxybacter	0.18 ± 0.18[a]	0 ± 0[a]	0.374	0.5538
Desulfovibrio	2.74 ± 1.67[a]	0.58 ± 0.58[a]	0.289	0.5538
Helicobacter	0 ± 0[a]	0.12 ± 0.12[a]	0.374	0.5538
Enterobacter	0.43 ± 0.43[a]	0 ± 0[a]	0.374	0.5538
Halomonas	0 ± 0[a]	0.07 ± 0.07[a]	0.374	0.5538
Actinobacillus	1.43 ± 1.35[a]	0.07 ± 0.05[a]	0.369	0.5538
Avibacterium	1.69 ± 1.61[a]	1.36 ± 1.14[a]	0.873	0.8963
Haemophilus	14.82 ± 14.67[a]	1.24 ± 0.41[a]	0.407	0.5698
Mannheimia	0.15 ± 0.15[a]	0 ± 0[a]	0.374	0.5538
Pasteurella	1.59 ± 1.37[a]	0.06 ± 0.06[a]	0.326	0.5538
Acinetobacter	0.68 ± 0.68[a]	0 ± 0[a]	0.374	0.5538
Pseudomonas	0.37 ± 0.27[a]	0 ± 0[a]	0.239	0.5538
Vibrio	0 ± 0[a]	0.09 ± 0.09[a]	0.374	0.5538
Treponema	0 ± 0[a]	0.83 ± 0.83[a]	0.374	0.5538
Candidatus.phytoplasma	0.17 ± 0.17[a]	0.06 ± 0.03[a]	0.563	0.6493
Mycoplasma	0.05 ± 0.05[a]	0 ± 0[a]	0.374	0.5538
Chromera	0.09 ± 0.09[a]	0 ± 0[a]	0.374	0.5538

Values are means ± SEM. [a-b] Means in a row without a common superscript letter differ ($P < 0.05$) as analyzed by one-way ANOVA and the TUKEY test.

Table S15: PLS-DA Genus VIP scores used for loadings name scatterplot of foregut samples

Bacteria	VIP
Desulfovibrio	3.816
Allobaculum	3.591
Gracilibacter	3.188
Christensenella	2.885
Lactobacillus	2.871
Yaniella	2.769
Halospirulina	2.381
Salinicoccus	2.225
Chromera	1.792
Streptococcus	1.775
Corynebacterium	1.671
Staphylococcus	1.576
Paracoccus	1.488
Gloeobacter	1.445
Ruegeria	1.441
Bacillus	1.333
Halomonas	1.217
Vibrio	1.173
Lachnoclostridium	1.080
Sporobacter	1.050
Rothia	1.031
Comamonas	1.011

Table S16. Alpha diversity metrics comparing colony and wild foregut microbiome samples

		Colony		Wild				
	n	mean	sd	mean	sd	t stat	p-value	fdr p-value
Chao1	6	524.03	116.99	632.31	205.51	-0.82	0.58	0.58
Faith's PD	6	27.54	6.27	35.19	8.46	-1.19	0.21	0.21
Goods coverage	6	1.00	0.00	1.00	0.00	0.86	0.42	0.42
Observed species	6	401.80	134.15	533.73	240.77	-0.86	0.47	0.47
Shannon	6	3.60	1.15	3.89	1.97	-0.25	1	1
Simpson	6	0.74	0.16	0.74	0.25	0.01	1	1
